# Validation of body composition parameters extracted via deep learning-based segmentation from routine computed tomographies

**DOI:** 10.1038/s41598-025-96238-6

**Published:** 2025-04-07

**Authors:** Felix O. Hofmann, Christian Heiliger, Tengis Tschaidse, Stefanie Jarmusch, Liv A. Auhage, Ughur Aghamaliyev, Alena B. Gesenhues, Tobias S. Schiergens, Hanno Niess, Matthias Ilmer, Jens Werner, Bernhard W. Renz

**Affiliations:** 1 Department of General, Visceral and Transplant Surgery, Ludwig-Maximilians-University Hospital Munich, Marchioninistrasse 15, 81377 Munich, Germany; 2 German Cancer Consortium (DKTK), Partner Site Munich, and German Cancer Research Center (DKFZ), Heidelberg, Germany; 3 Department of Radiology, Ludwig-Maximilians-University Hospital Munich, Munich, Germany

**Keywords:** Body composition, Computed tomography, Sarcopenia, Tissue segmentation, Oncology, Prognostic markers, Surgical oncology, Pancreatic cancer, Colorectal cancer

## Abstract

Sarcopenia and body composition metrics are strongly associated with patient outcomes. In this study, we developed and validated a flexible, open-access pipeline integrating available deep learning-based segmentation models with pre- and postprocessing steps to extract body composition measures from routine computed tomography (CT) scans. In 337 surgical oncology patients, total skeletal muscle tissue (SM_total_), psoas muscle tissue (SM_psoas_), visceral adipose tissue (VAT), and subcutaneous adipose tissue (SAT) were quantified both manually and using the pipeline. Automated and manual measurements showed strong correlations (SM_psoas_: r = 0.776, VAT: r = 0.993, SAT: r = 0.984; all *P* < 0.001). Measurement discrepancies primarily resulted from segmentation errors, anatomical anomalies or image irregularities. SM_psoas_ measurements showed substantial variability depending on slice selection, whereas SM_total_, averaged across all L3 levels, provided greater measurement stability. Overall, SM_total_ performed comparably to SM_psoas_ in predicting overall survival (OS). In summary, body composition measures derived from the pipeline strongly correlated with manual measurements and were prognostic for OS. The increased stability of SM_total_ across vertebral levels suggests it may serve as a more reliable alternative to psoas-based assessments. Future studies should address the identified areas of improvement to enhance the accuracy of automated segmentation models.

## Introduction

Sarcopenia, characterized by the loss of skeletal muscle mass, has gained significant relevance in both oncology and oncological surgery^1–4^. Affecting approximately 35% of oncology patients^5^, it is associated with poorer overall survival (OS), reduced disease-free survival, and diminished response to cancer therapies^2,6,7^. In surgical contexts, it is consistently linked to higher risk of postoperative complications^8–10^. For instance, in pancreatic cancer, sarcopenia is associated with higher perioperative mortality and shorter OS^11^. Early recognition of sarcopenia is therefore critical, as it allows targeted prehabilitation strategies—such as structured exercise programs and nutritional optimization—that may improve both short- and long-term outcomes^11,12^. Identifying sarcopenic patients before surgery provides an opportunity for timely intervention, potentially mitigating the clinical impact of muscle loss and improving patient prognosis.

Sarcopenia or its surrogate parameters can be determined using various methods, such as dual-energy X-ray absorptiometry, bioelectrical impedance analysis, hand grip tests, clinical scores, or the analysis of imaging studies^4^. The evaluation of computed tomography (CT) scans is particularly well established due to its widespread availability and the standardized, reproducible nature of measurements^4,13^. However, manual measurements are labor-intensive, limiting their practicality in clinical routine.

Given the advances in deep learning-based semantic segmentation techniques, applying such methods to determine body composition parameters is promising. Several studies have demonstrated the feasibility of using deep learning models to segment muscle and adipose tissue areas, showing strong correlations with manual measurements and clinical outcomes^14–19^. However, widespread adoption of these models is hampered by limited accessibility^15,20,21^.

In response, we developed a flexible pipeline that integrates existing segmentation models with pre- and postprocessing steps to assess sarcopenia and body composition measures. This study aimed to validate this automated pipeline against manual measurements in a real-world cohort of surgical oncology patients, with particular emphasis on identifying sources of discrepancy and assessing measurement reliability across different skeletal muscle metrics.

## Materials and methods

### Study design

**Fig. 1:**
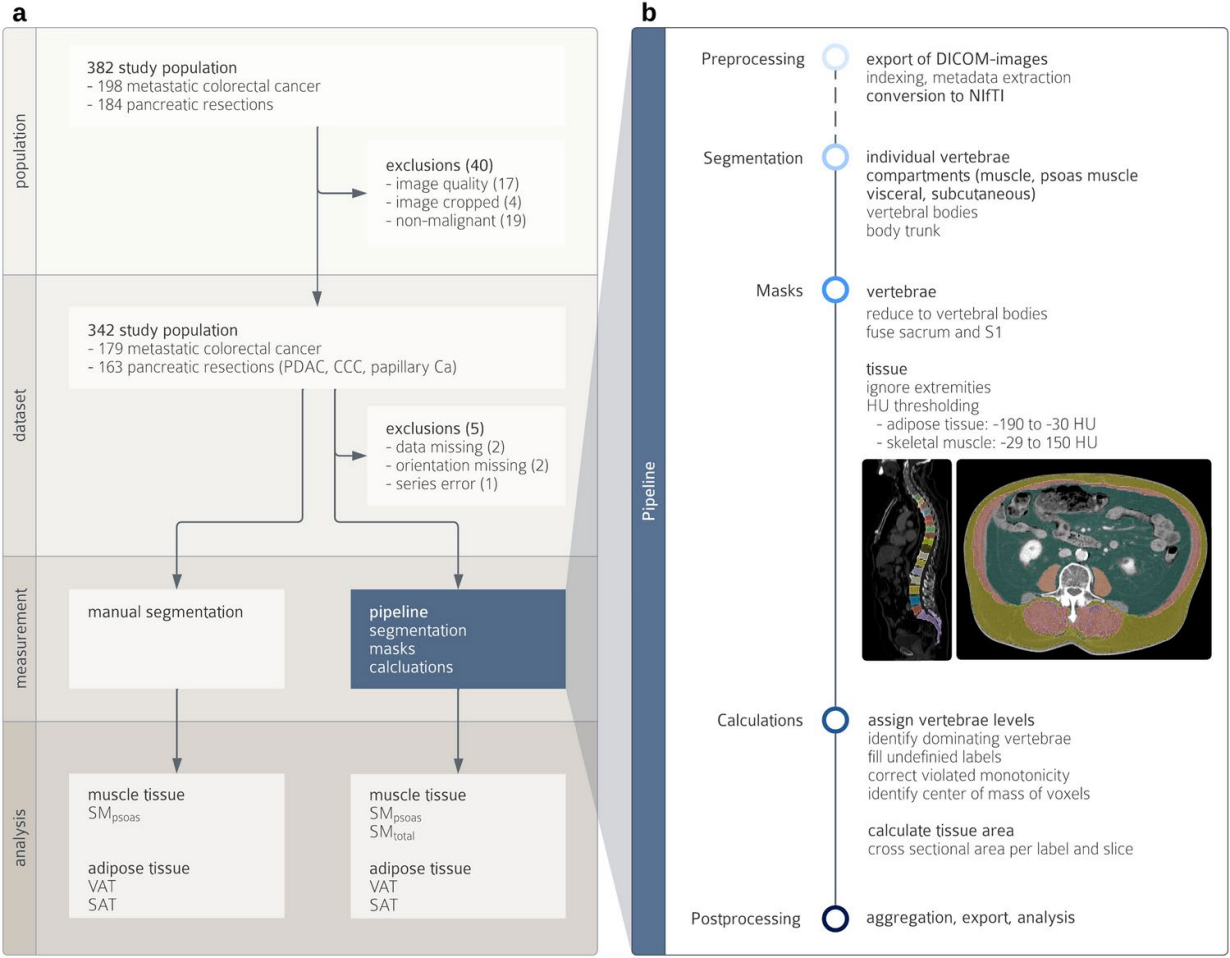
Study design (**a**) and segmentation pipeline (**b**): Manual and deep learning-based segmentations and postprocessing steps to measure cross sectional areas (CSA) of muscle tissue (SM_psoas_, SM_total_), visceral adipose tissue (VAT), and subcutaneous adipose tissue (SAT).

This study is a non-interventional, retrospective analysis of 382 patients who underwent surgery for hepatic or pancreatic disease between 2003 and 2016 (Fig. 1a). We included patients with underlying oncological disease and available CT scans of adequate quality and coverage performed up to two months prior to surgery (see Online Resource 1, Image Quality Criteria). Manual measurements were derived from earlier, unpublished analyses. Five patients were secondarily excluded from deep learning-based measurements due to missing imaging data (n = 2), metadata errors (n = 2) preventing accurate image orientation, or incomplete imaging data (n = 1) caused by series reconstruction issues (see Online Resource 2, Secondary Exclusions). These exclusions were unrelated to segmentation accuracy but based on data availability only.

The study received ethical approval from the institutional review board (IRB) of the medical faculty of the LMU Munich (23-0891) and was registered at the Clinical Study Center of the LMU University Hospital (102402). Data screening was performed by authors who were obligated to confidentiality. CT scans, OS and body height of the included patients were irreversibly anonymized, and all analyses were conducted thereafter. The IRB determined that informed consent was not necessary, as all data utilized in the study were initially collected for clinical purposes, the study design was entirely retrospective, and all analyses were performed only in the irreversibly anonymized dataset.

### Imaging

CT scans were performed either at LMU University Hospital or at referring institutions, using a variety of CT scanners from four manufacturers. A weight-adapted dose of iodinated contrast agent was administered intravenously in all cases, and portal venous phase scans were preferred for analysis. Image data were reconstructed using a standard soft tissue kernel with a maximum slice thickness of 8 mm.

### Definitions

In this study, we distinguished between *compartments* and *tissue* (Table 1). *Compartments* are defined by anatomical boundaries such as skin, fascia, bone, vessels, and organs. *Tissue* refers to the actual tissue composition within each compartment and was assigned based on Hounsfield Units (HU) using established thresholds^3,4,7,17^. We determined the cross-sectional area (CSA) of total skeletal muscle tissue (SM_total_), psoas muscle tissue (SM_psoas_), visceral adipose tissue (VAT), and subcutaneous adipose tissue (SAT).

**Table 1:** Definitions of compartments and tissue.

Compartment		Thresholds	Tissue
Label	Definition	HU	Label
Muscle	Regions predominantly including muscles, without separating individual muscles. Includes smaller vessels and inter- and intramuscular connective tissues	− 29 to 150	Total skeletal muscle tissue (SM_total_)
Iliopsoas Muscle	Subset of muscle compartment, including only iliopsoas muscle	− 29 to 150	Psoas skeletal muscle tissue (SM_psoas_)
Visceral	Regions beneath the thoracic cage and muscles of the abdominal wall. Includes pelvis and retroperitoneum. Excludes solid organs and larger vessels	− 190 to − 30	Visceral adipose tissue (VAT)
Subcutaneous	Regions beneath the skin, overlaying fascias or periosteum. Excludes foreign material (e.g., implants)	− 190 to − 30	Subcutaneous adipose tissue (SAT)

Previous studies have demonstrated that the CSA of total muscle at the third lumbar vertebra (L3) correlates well with overall muscle mass^4,13^. Some authors have used psoas muscle CSA as a simplified surrogate for total muscle mass^22–24^, although this approach remains under debate^22,25,26^. Therefore, the pipeline was constructed to determine deep learning-based measurements for both SM_total_ and SM_psoas_.

### Manual measurement

Manual measurements were obtained from earlier, unpublished analyses. They were performed using the SliceOmatic software (version 5.0; Tomovision, Magog, Quebec, Canada) by three readers (2 medical students under supervision of CH) under the guidance of a board-certified radiologist (ABG). First, the first lumbar vertebra (L1) was identified as the most cranial lumbar vertebra without rib attachments. The readers then counted downward to locate L3. The axial slice at L3 where both transverse processes were clearly visible was selected for analysis. In this slice, standard HU thresholds were applied to measure the respective *tissue* CSA. Segmentation was performed using either manually marking the relevant areas (“Paint method”) or selecting a single pixel and automatically including all connected pixels (“Grow 2D method”).

### Automated measurement

Automated measurements were performed by mirroring the manual measurement workflow (Fig. 1b). The developed pipeline integrates TotalSegmentator^27^, with specific pre- and postprocessing steps (Fig. 1b). The pipeline’s source code and detailed documentation are available at https://github.com/fohofmann/BodyComposition/releases/tag/v0.1.

CT scans of the included patients were exported with standardized image formatting of 512 × 512 voxels, and the DICOM files were converted to NIfTI format. First, segmentations of the spine (vertebrae T1 to L5, S1 and os sacrum) and compartments (muscle, visceral, and subcutaneous) were performed using TotalSegmentator. Segmentations of the body trunk, psoas muscle, and vertebral bodies (excluding the spinous and transverse processes) were created separately. Using the latter, labels of the spine were reduced to include only the vertebral bodies, excluding the vertebral arch and spinous processes. The center of each vertebral body was then determined by calculating the center of mass of the assigned voxels. Compartment labels were confined to the body trunk, excluding extremities, head and neck. Subsequently, the tissue composition of the specific compartments was determined using established thresholds (Table 1). For each CT slice and tissue type, the CSA was calculated.

### Qualitative and statistical analysis

The relationship between manual and automated measurements of tissue CSA was assessed using Bland–Altman analysis and correlation metrics. Since manual segmentations were not systematically stored in a format enabling voxel-wise comparison, calculation of the Dice similarity coefficient was not feasible. In a subset of patients who underwent pancreatic surgery, measurement inconsistencies and outliers (defined as deviations beyond the 95% confidence interval) were qualitatively explored, categorized, and described. The colorectal cancer cohort was not included in the discrepancy analysis, as manual measurements lacked documentation regarding the specific slice selection. Exemplary visualizations were created using 3D slicer, integrating labels obtained through manual HU thresholding with those derived from the pipeline^28^. To assess measurement variability across vertebral levels, cases with segmentation errors in L2, L3 or L4 were excluded, and CSA values (per slice and per vertebra) at L2 and L4 were compared to their respective reference at L3. A linear mixed effects model was developed, including the vertebral level as a fixed effect and a random intercept for each patient. The median overall survival (OS) of patients with pancreatic or colorectal cancer was assessed using Kaplan–Meier analysis. The skeletal muscle index (SMI = CSA muscle/(body height [m])^2^) was calculated^2^. For each entity, the association of SMI with OS was analyzed using Harrell’s C-index and receiver operating characteristic (ROC) analysis to identify patients with above-median OS, as well as Kaplan–Meier survival analyses. Hazard ratios (HR) with 95% confidence intervals were calculated using Cox proportional hazards regression. All statistical analyses were performed using R version 4.4.0. The significance level was set at 0.05, and all tests were conducted two-sided.

## Results

### Patient characteristics

This study included 337 patients who underwent oncological surgery. Among these patients, 174/337 (51.6%) had metastatic colorectal cancer, 150/337 (44.5%) had pancreatic ductal adenocarcinoma, 7/337 (2.1%) had carcinoma of the papilla of vater, and 6/337 (1.8%) had extrahepatic cholangiocarcinoma. Of the included patients, 140/337 (41.5%) were female. The age of the patients ranged between 21 and 85 years, with a mean age of 64.5 years (standard deviation, 10.8 years).

CT scans were performed a median of 15 days prior to surgery (interquartile range [IQR], 5 to 29 days). Slice thickness ranged from 0.5 to 8 mm, with a median slice thickness of 5 mm (IQR, 3 to 5 mm). The median number of slices per CT scan was 129 (IQR, 90 to 195).

### Deep learning-based vs. manual measurements

**Fig. 2:**
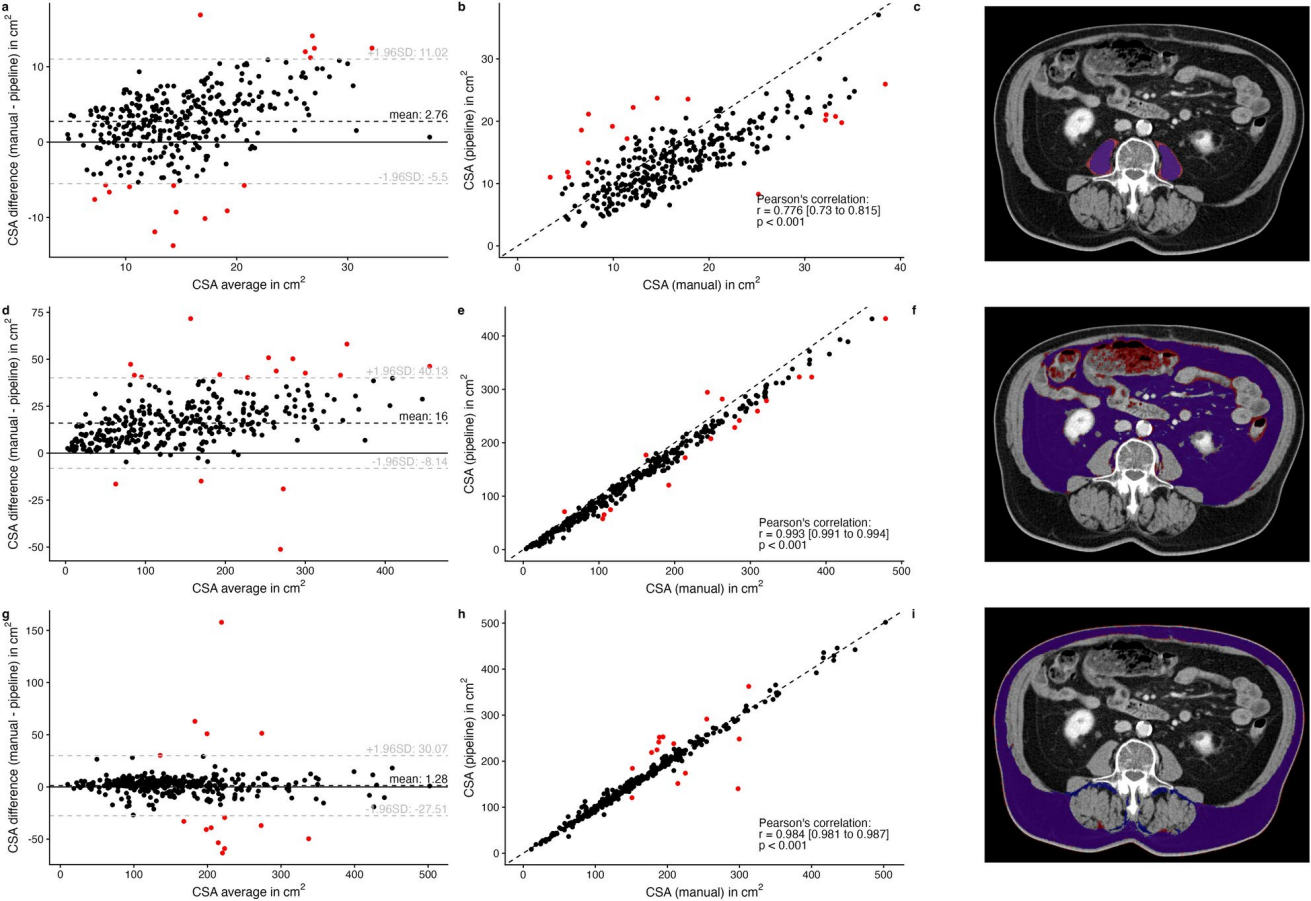
Comparison of manual and automated measurements: Bland–Altman plots (**a**, **d**, **g**) showing the differences between cross-sectional area (CSA) measurements for the psoas muscle tissue (**a**, **b**, **c**), visceral adipose tissue (**d**, **e**, **f**), and subcutaneous adipose tissue (**g**, **h**, **i**), with outliers marked in red; Scatter plots (**b**, **e**, **h**) illustrating the respective correlations; Example images of the respective segmentations (**c**, **f**, **i**) with manual segmentations (red), deep learning-based segmentations (blue), and intersections (purple).

At the level of L3, Bland–Altman analysis indicated that the CSA of SM_psoas_ and VAT was lower when measured using the pipeline compared to manual measurements (Fig. 2a,d). In contrast, no relevant systematic difference was observed between manual and automated measurements for the CSA of SAT (Fig. 2g). All measurements correlated strongly or very strongly (SM_psoas_ r = 0.776, 95% CI 0.730 to 0.815; *P* < 0.001; VAT r = 0.993, 95% CI 0.991 to 0.994; *P* < 0.001; SAT r = 0.984, 95% CI 0.981 to 0.987; *P* < 0.001) (Fig. 2b,e,h). Exemplary segmentations are shown in Fig. 2c,f,i.

### Causes of measurement discrepancies

**Fig. 3:**
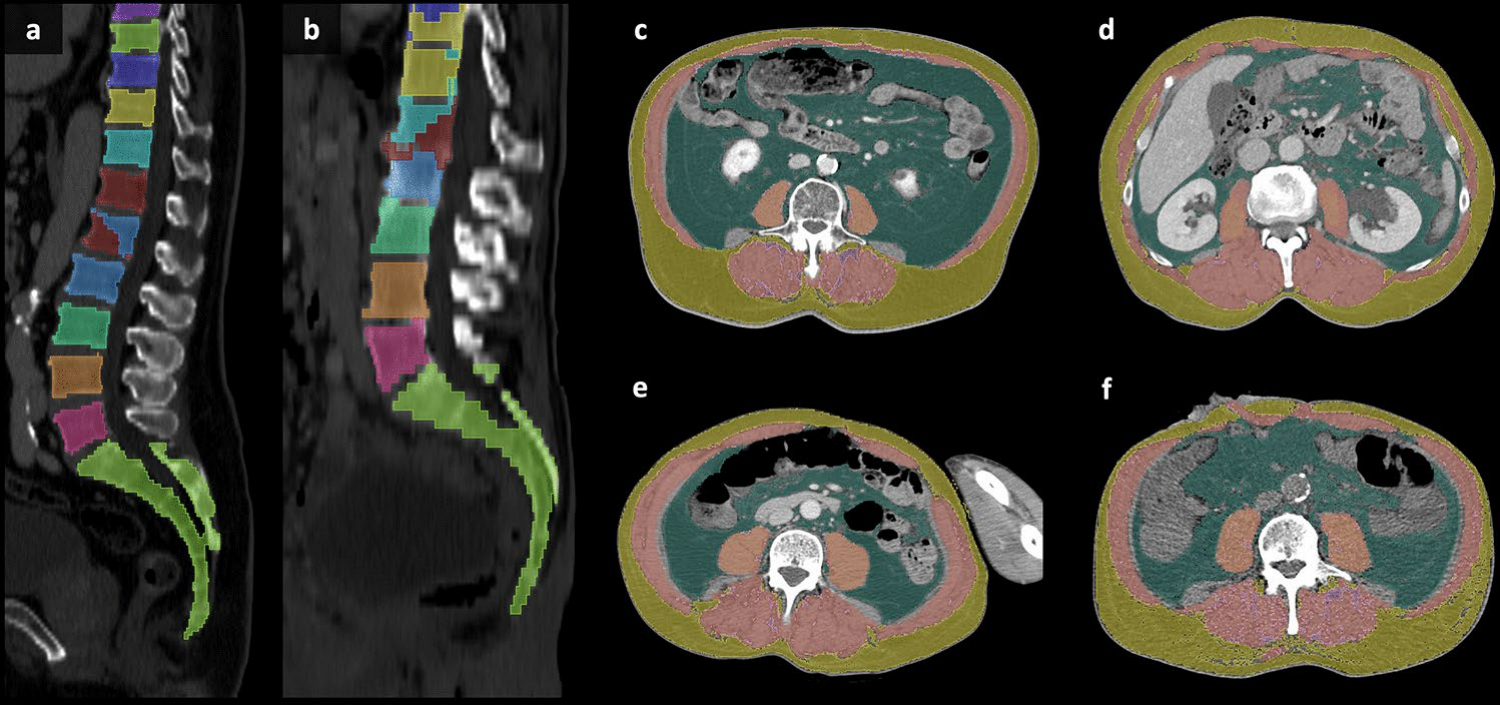
Examples of deep learning-based segmentation errors: segmentation errors of the vertebral bodies (**a**, **b**), caused by vertebral anomalies (**a**, lumbarization of S1) or by factors not associated with anomalies (**b**); Segmentation errors of tissue (**d**–**f**), including inaccurate segmentation of specific muscle groups (**c**, **d**), included extremities (**e**, corrected), and noisy CT scans (**f**); skeletal muscle tissue delineated in red, visceral adipose tissue in green, subcutaneous adipose tissue in yellow, and intermuscular adipose tissue in pink.

In a subset of patients who underwent pancreatic surgery, the levels identified as the center of L3 by the manual reader and the pipeline were compared. In 150/163 (92.0%) patients, the same or directly neighboring slices were identified. In 4 cases (2.4%), the level differed by two or three slices but was still within the L3 vertebral level. In 8 cases (4.9%) the deep learning-based segmentations included errors, with some errors associated with vertebral anomalies (e.g., sacralization of lumbar vertebrae or lumbarization of sacral vertebrae, Fig. 3a), and others occurring independently of any anomalies (Fig. 3b) (see Online Resource 3, Causes of Measurement Differences).

The CSA of VAT measured using the pipeline was lower compared to manual measurements. This discrepancy was primarily due to the overestimation that occurred when voxels within the abdominal cavity were selected as VAT based solely on HU thresholds, leading to the misclassification of structures such as bowel contents (Fig. 2f). SM_psoas_ measurements showed greater variability between manual and automated measurements, whereas SAT and VAT demonstrated more consistent correlations (Fig. 2). In the analysis of all outliers, causes of deviations included mislabeling of the quadratus lumborum muscle (Fig. 3c), the abdominal wall (Fig. 3c) or the psoas muscle (Fig. 3d), inclusion of extremities in the analyzed slice (Fig. 3e), or noisy CT scans (Fig. 3f).

### Influence of the imaging plane on measurements

**Fig. 4:**
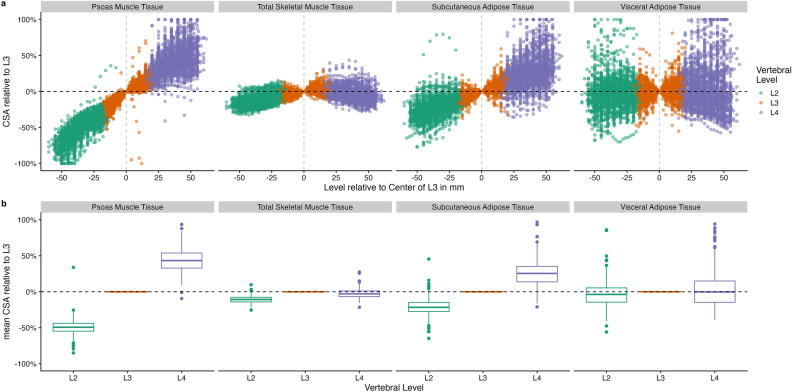
Cross-sectional areas (CSA) per slice relative to the reference measurement obtained at the center of L3 (**a**) for the psoas muscle tissue, total skeletal muscle tissue, subcutaneous adipose tissue, and visceral adipose tissue with negative and positive values indicating measurements taken below and above the center of L3, respectively; mean CSA of the respective tissue type (**b**) across different vertebral levels (L2, L3, and L4) relative to L3.

To assess the impact of the selected slice, the automatically determined CSA per slice was compared to reference measurements obtained at the center of L3. CSA of SM_psoas_ and SAT were larger in the lower lumbar spine (Fig. 4a), whereas no similar trend was observed for SM_total_ and VAT. The extent of deviation for all measurements progressively increased with the distance from the reference level at the center of L3. Measurements taken just one slice above or below the reference level, but still within the L3 vertebra, exhibited deviations of up to 50% in some cases. Notably, SM_total_ showed greater stability across different measurement levels (Fig. 4a).

### Reducing measurement variability

To address the variability of measurements across different slices, we computed the mean CSA per vertebral level and compared the values at L2 and L4 relative to L3 (Fig. 4b). SM_psoas_ and SAT were consistently greater at lower levels, whereas no systematic trend was observed for the SM_total_ and VAT. The influence of vertebral level on measurement variability was explored using a linear mixed-effects model. For SM_psoas_, the fixed effect estimates showed a significantly lower CSA at L2, and a significantly higher CSA at L4 compared to L3. A similar, but less pronounced trend was observed for SAT. For SM_total_, the CSA was lower at both L2 and L4 compared to L3, with smaller relative differences (Table 2).

**Table 2:** Mixed effects model.

Tissue type	Fixed effect	CSA estimate [cm^2^]	SE	*p*-value	CSA estimate relative to L3 [%]
SM_psoas_	L3 (Reference)	13.7	0.3	*p* < 0.001	
	L2	− 6.6	0.1	*p* < 0.001	52.1%
	L4	5.7	0.1	*p* < 0.001	141.8%
SM_total_	L3 (Reference)	114.8	1.5	*p* < 0.001	
	L2	− 12.3	0.4	*p* < 0.001	89.3%
	L4	− 3.2	0.4	*p* < 0.001	97.2%
VAT	L3 (Reference)	136.4	4.9	*p* < 0.001	
	L2	− 5.3	1.6	*p* < 0.001	96.1%
	L4	− 7.0	1.6	*p* < 0.001	94.9%
SAT	L3 (Reference)	162.3	4.9	*p* < 0.001	
	L2	− 31.0	1.5	*p* < 0.001	80.9%
	L4	35.4	1.5	*p* < 0.001	121.8%

Fixed effect estimates, standard errors, *p*-values, and relative values for the mean cross-sectional area (CSA) of the psoas skeletal muscle tissue (SM_psoas_), total skeletal muscle tissue (SM_total_), visceral adipose tissue (VAT) and subcutaneous adipose tissue (SAT) at different vertebral levels, relative to L3.

### Association with overall survival

**Fig. 5:**
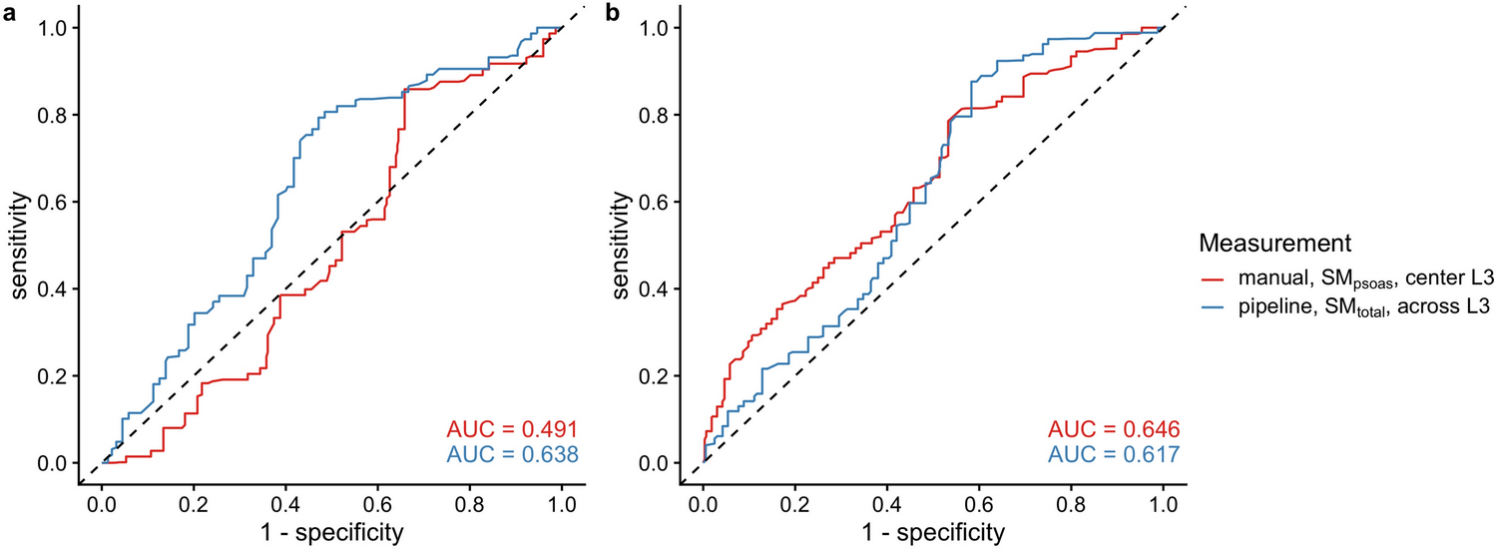
ROC analysis of skeletal muscle index (SMI) predicting above-median overall survival (OS) for (**a**) patients with pancreatic cancer (> 19.9 months [95% CI, 16.0 to 23.0 months]), and (**b**) patients with metastatic colorectal cancer (> 47.9 months [95% CI, 40.7 to 61.0 months]). The red line represents SMI derived from manual psoas muscle measurements (SM_psoas_) at the center of L3, while the blue line represents SMI based on total skeletal muscle tissue (SM_total_) averaged across all levels of L3 as determined by the pipeline.

**Fig. 6:**
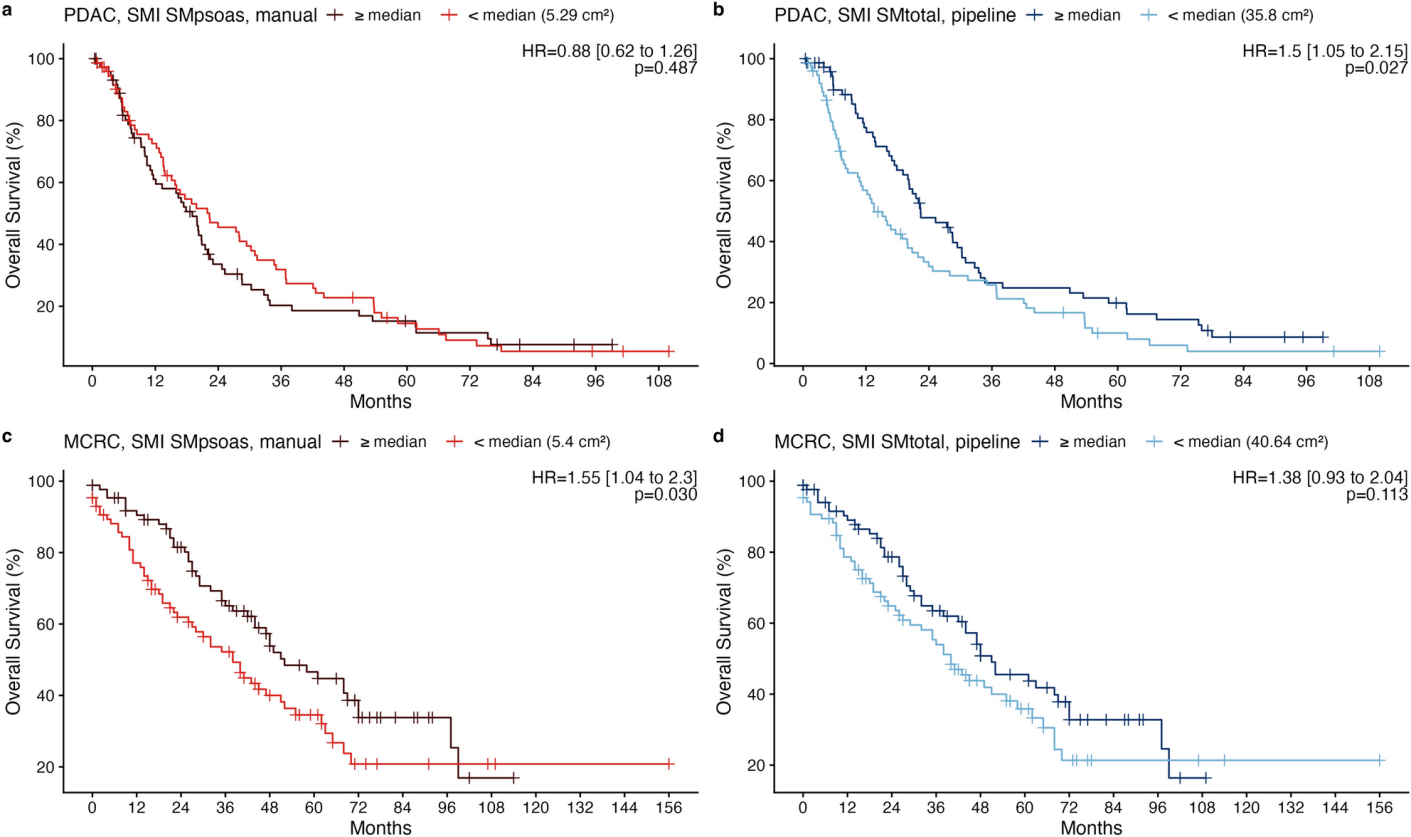
Kaplan–Meier survival curves illustrating overall survival (OS) in patients with pancreatic cancer (**a**, **b**) and metastatic colorectal cancer (**c**, **d**), stratified by skeletal muscle index (SMI) derived from manual psoas muscle measurements (SM_psoas_) at the center of L3 (**a**, **c**) or based on pipeline-derived total skeletal muscle tissue (SM_total_) averaged across L3 (**b**, **d**). Patients were grouped based on the median SMI within each cohort and measurement method. Hazard ratios (HR) and *p*-values were determined using Cox proportional hazard regression analysis.

We compared the prognostic relevance of the SMI derived from manual psoas muscle measurements SM_psoas_ at the L3 center and automated total skeletal muscle measurements SM_total_ averaged across L3: In patients with pancreatic cancer, the SM_total_-based SMI demonstrated significantly higher prognostic accuracy than SM_psoas_-based SMI (C-index 0.598 vs. 0.494; *P* < 0.001). Additionally, the AUC in ROC analysis for predicting above-median OS was higher for automated SM_total_-based SMI compared to manual SM_psoas_-based SMI (Fig. 5a). In patients with colorectal cancer, prognostic accuracy was comparable between the two methods, with no significant difference in C-Index (0.579 vs. 0.626; *P* = 0.111). The AUC for automated SM_total_-based SMI was slightly lower than for manual SM_psoas_-based SMI (Fig. 5b). Kaplan–Meier survival analyses further illustrated the differences in OS between high- and low-SMI groups, with similar variations depending on the measurement method and cancer type (Fig. 6).

## Discussion

In this study, we developed a software pipeline that integrates pre- and postprocessing steps with open-access segmentation models to automate the measurement of body composition parameters from routine CT scans. In a cohort of 337 surgical-oncological patients, we assessed the concordance of automated measurements with manual measurements originating from earlier, unpublished analyses, explored potential causes of discrepancies, and investigated strategies to compensate for measurement errors.

We found a strong correlation between manual and automated measurements of subcutaneous and visceral adipose tissue (SAT, VAT) at the level of L3, with no relevant systematic differences. However, the concordance between manual and automated measurements of the psoas muscle tissue (SM_psoas_) was less consistent. Errors related to vertebral anomalies, such as lumbarization of S1 or sacralization of L5, occasionally resulted in incorrect selection of L3 by the pipeline^29^, or muscle tissue was not correctly segmented. Additionally, noisy CT scans and the proximity of tissue to structures with similar HU values complicated threshold-based sub-segmentations^21^.

We observed that the CSA of SM_psoas_ and SAT varied significantly depending on the slice selected for tissue segmentation. In contrast, SM_total_ remained more stable across vertebral levels, making it a more robust and reproducible marker for skeletal muscle mass. By averaging SM_total_ across the entire L3 vertebra, segmentation inconsistencies are mitigated, and measurement reliability is improved. In this study, the mean SM_total_ across the entire L3 vertebra, as determined by the pipeline, showed comparable predictive value for above-median OS compared to manually determined SM_psoas_ at the center of L3.

Other groups have reported on individual deep learning-based tissue segmentation tools, but common issues include small datasets, unavailable training labels, restricted model weights, and a lack of clinical validation^14,15,18^. A recent study claimed that their model outperformed TotalSegmentator in tissue segmentation; however, labels or model weights were not provided^21^. Blankemeier et al. developed a pipeline that includes tissue segmentation, but the model is based on a 2D nnU-Net and was not evaluated clinically^16^. The Body and Organ Analysis (BOA) tool offers a comprehensive pipeline, including model weights^30^ and the underlying training labels^31^, and the association of its measurements with OS has been documented^32^. However, BOA uses the vertebral levels predicted by TotalSegmentator, and thus some limitations (and mitigation strategies) described in this study apply to BOA as well.

It is important to note that manual segmentation methods are not without errors. For instance, in the subset of patients who underwent pancreatic surgery, 2 of 13 discrepancies between manual and automated selection of the axial slice were caused by human errors. Moreover, a purely HU-based segmentation approach can lead to additional inaccuracies, particularly in noisy CT scans. Manual segmentation is prone to observer fatigue, particularly in repetitive tasks, whereas deep learning models offer scalability and can further improve by addressing identified weaknesses, for instance with refined training and postprocessing adjustments.

One limitation of this study is the absence of a pixel-by-pixel ground truth approximation. Since manual segmentations were not systematically stored in a format enabling voxel-wise comparisons, a Dice similarity coefficient could not be calculated. Although the Dice score is a widely used metric for segmentation accuracy, it does not necessarily correlate with clinical relevance^15^, and we therefore focused on evaluating whether body composition measures derived from the deep learning-based pipeline are comparable to manually obtained measures. While this study discusses issues arising from segmentation errors, it does not provide improved training labels or propose new models. Future work could build on openly available datasets to improve labels, particularly for the segmentation of vertebral bodies with anatomical anomalies. This could enhance the accuracy and reliability of automated segmentation tools, addressing some of the limitations we identified.

The prognostic value of the SMI was limited in this study, particularly in the ROC-analysis of patients with metastatic colorectal cancer. This limitation could stem from the multifactorial nature of oncological patient outcomes, which the SMI alone may not fully capture. Additionally, using above-median OS as an evaluation metric might not be ideal for patients with longer survival, as sarcopenia at the time of diagnosis could more significantly impact short-term outcomes^33^.

Although this study focused on pancreatic and metastasized colorectal malignancies due to data availability, the published pipeline itself is disease-agnostic and can be applied across oncological and non-oncological conditions. Future studies should explore the prognostic value of different measurement techniques across various patient groups and cancer types. A deeper understanding of body composition and its changes could be used to optimize the combination, timing, and intensity of multimodal therapy for individual cancer patients.

In summary, our open-access pipeline integrates established deep learning segmentation models with postprocessing steps to extract body composition measures from routine CT scans. Automated measurements showed strong correlation with manual measurements and were prognostic for overall survival. Averaging CSA across the entire L3 vertebra minimized measurement variability, reinforcing SM_total_ as a robust and reliable marker for sarcopenia assessment. Our study highlights the clinical potential of automated segmentation tools while identifying key areas for future improvement and validation.

## Supplementary Information

Supplementary Information.

## Data Availability

The clinical data and CT scans analyzed this study cannot be shared due to data protection laws. The source code and a docker image of the pipeline are available under Apache 2.0 license at https://github.com/fohofmann/BodyComposition/releases/tag/v0.1. The segmentation tasks are performed using TotalSegmentator, available at https://github.com/wasserth/TotalSegmentator under Apache 2.0 license^27^. The weights for the segmentation models were available under free or non-commercial usage licenses from https://github.com/wasserth/TotalSegmentator. TotalSegmentator uses the nnU-Net framework^34^.
