# Supplementary material for: Validation of body composition parameters extracted via deep learning-based segmentation from routine computed tomographies
Source: Sci Rep. 2025 Apr 7;15:11909. doi: 10.1038/s41598-025-96238-6 (PMC11977262; doi:10.1038/s41598-025-96238-6)
Supplement: Supplementary file 1 — Supplementary Information. [file 41598_2025_96238_MOESM1_ESM.pdf]

Supplementary Information I

Article	Validation of Body Composition Parameters Extracted via Deep Learning-Based Segmentation from Routine Computed Tomographies
Journal	Scientific Reports
Authors	Felix O. Hofmann, Christian Heiliger, Tengis Tschaidse, Stefanie Jarmusch, Liv A. Auhage, Ughur Aghamaliyev, Alena B. Gesenhues, Tobias S. Schiergens, Hanno Nieß, Matthias Ilmer, Jens Werner, Bernhard W. Renz
Corresponding Author	Felix O. Hofmann, MD, M.Sc.<br>Department of General, Visceral and Transplantation Surgery<br>University Hospital, LMU Munich<br>Marchioninistrasse 15, 81377 Munich, Germany<br>Tel.: +49-89-4400-0<br>ORCID: 0000-0002-6913-2429<br>Email: Felix.Hofmann@med.uni-muenchen.de

Online Resource 1: Imaging Quality Criteria

We included computed tomography (CT) scans of adequate quality and coverage, defined as follows:

- **Slice Thickness**: A maximum slice thickness of 8 mm to ensure sufficient resolution for identifying and segmenting vertebrae (and soft tissue).
- **Contrast Phase**: A contrast-enhanced phase was required (portal venous phase preferred) to better distinguish different tissue types.
- **Coverage of L2–L4**: Full visibility of the L2–L4 vertebrae was essential, as L3 served as the landmark for measuring the body composition parameters.
- **Subcutaneous Compartment**: The entire body, especially the complete subcutaneous tissue, needed to be included in the selected axial slice to enable accurate quantification.

These criteria were deliberately kept broad to allow the inclusion of scans from both our own center and referring institutions. Most excluded patients were not omitted for failing these requirements but rather due to the unavailability of any CT scan, especially in the earlier years covered by the study.

Online Resource 2: Secondary Exclusions

Five patients with existing manual measurements could not be processed by the automated pipeline due to following reasons:

- **Missing imaging data (n=2):** We could not verify which CT scans were used by the manual readers, as none of the available studies met the inclusion window (up to two months before surgery).
- **Metadata Errors (n=2):** Significant errors in the DICOM metadata regarding the orientation and scaling made reorientation and segmentation impossible.
- **Missing Slices (n=1):** Multiple imaging series were merged into a single series, however crucial slices remained missing, preventing complete segmentation.

Online Resource 3: Causes of Measurement Differences

In the subgroup of patients who underwent pancreatic surgery, the levels identified as the center of L3 by the manual reader and the automated workflow were compared. In 150 out of 163 patients (92.0%), the pipeline identified the same or directly neighboring slices as the manual reader.

The remaining 13 out of 163 patients (8.0%) were analyzed individually to determine the underlying causes of discrepancies (Supplementary Table 1). The causes were:

- In 4 cases (2.4%), the level differed by two or three slices but was still within the L3 vertebral level.
- In 6 cases (3.7%), the deep-learning-based segmentations were erroneous, likely associated with vertebral anomalies (e.g., sacralization of lumbar vertebrae or lumbarization of sacral vertebrae) (Fig. 3a).
- In 1 case (0.6%), the human reader misidentified L4 as L3.

- In 1 case (0.6%), the human reader misidentified L2 as L3, and the deep learning-based segmentation included an error without the presence of relevant vertebral anomalies (Fig. 3b).
- In 1 case (0.6%), the deep-learning based segmentation included errors, most likely due to vertebral anomalies, which affected the postprocessing algorithm's ability to identify the centroid.

Patient-ID	Slice Difference: manual vs. automated workflow (number of slices / mm)	Error
CCC-236	2 / 6	no segmentation error, different slice at L3 level
PDAC-83	-6 / -30	segmentation error T11 - L2 without vertebral anomalies<br>human reader error (L2 identified as L3)
PDAC-125	-3 / -15	segmentation error T10 - L3 with vertebral anomalies<br>rarefied ribs at T12 ⟶ labeled as L1
PDAC-126	-6 / -30	segmentation error L2 - Sacrum with vertebral anomalies:<br>sacralized L5 ⟶ labeled as S1
PDAC-153	6 / 24	segmentation error L3 - Sacrum with vertebral anomalies:<br>lumbarized S1 ⟶ labeled as L5
PDAC-156	NA	segmentation error L2 - Sacrum with vertebral anomalies:<br>sacralized L5 ⟶ labeled as S1
PDAC-181	-2 / -8	no segmentation error, different slice at L3 level
PDAC-197	3 / 9	no segmentation error, different slice at L3 level
PDAC-259	11 / 33	segmentation error L1 - Sacrum with vertebral anomalies:<br>lumbarized S1 ⟶ labeled as L5
PDAC-275	-4 / -20	segmentation error T10 - Sacrum with vertebral anomalies:<br>sacralized L5 ⟶ labeled as S1
PDAC-356	-18 / -36	human reader error (L4 identified as L3)
PDAC-360	-4 / -20	segmentation error T12 - L3 with vertebral anomalies<br>rarefied ribs at T12 ⟶ labeled as L1
PapillaryCa-93	-2 / -4	no segmentation error, different slice at L3 level

Supplementary Table 1: Reasons for discrepancies in the image plane selected for measurement between deep learning-based segmentation with postprocessing and manual selection. Slices were numbered from feet to head; thus, positive numbers indicate that the deep learning-based selected slice was closer to the feet than the manually selected slice.
